# A Study of 279 General Outbreaks of Gastrointestinal Infection in the North-East Region of England

**DOI:** 10.3390/ijerph6020547

**Published:** 2009-02-05

**Authors:** Grahame M. Tebbutt, Deborah Wilson, Ian Holtby

**Affiliations:** 1 HPA North East, Newcastle Laboratory, Institute of Pathology, Newcastle General Hospital, Newcastle-upon-Tyne NE4 6BE, UK; 2 County Durham and Tees Valley Health Protection Unit, Appleton House, Lanchester Road, Durham DH1 5XZ, UK; E-Mail: Deborah.Wilson@hpa.org.uk; 3 County Durham and Tees Valley Health Protection Unit, Poole House, Stokesley Road, Middlesbrough TS7 0NJ, UK; E-Mail: Ian.Holtby@hpa.org.uk

**Keywords:** Foodborne disease, outbreak, surveillance

## Abstract

All outbreaks of infectious intestinal disease reported to the authorities were entered on a computer database with outbreak control teams being established to investigate larger or more significant incidents. The outbreak database and, when set up, the notes of outbreak team meetings were examined for the 279 outbreaks reported in a three-year period (2003–2005). Faeces specimens submitted as part of an outbreak were examined for microbial pathogens and the results cross-matched to the outbreak number. Almost half of the general outbreaks reported (137) occurred in long-term care facilities for the elderly, 51 outbreaks were recorded in hospitals and 31 occurred in the wider community. In 76 outbreaks no specimen was logged. A microbial cause was confirmed in about one-third of outbreaks, with noroviruses being the most common (19%). *Salmonellas* accounted for 12 of the 21 community outbreaks linked to social events and all were foodborne. Suggestions for improving notification and surveillance are discussed.

## 1. Introduction

Infectious intestinal disease is common and the majority of cases go unreported. One study, based on 70 general practices across England, found that 1 in 6 patients presented to their general practitioner and that only a small proportion of these had pathogens isolated and were captured by national laboratory surveillance [1]. Larger outbreaks and those with more severe illnesses are more likely to be reported, and smaller incidents of mild disease or infections that spread slowly in a community may well be missed [2]. In the UK surveillance is largely based on laboratory confirmed cases where isolates have been sent to the reference laboratory for further typing and clusters have been identified. Surveillance of *Salmonella* and Verocytotoxin-producing *Escherichia* coli O157 infections based on laboratory isolates is now established (Enter-net) and this facilitates the investigation of international outbreaks caused by these pathogens [3].

Although most cases of gastrointestinal infection are self-limiting, some, particularly in the very young or old and those with underlying disease can be serious and even life-threatening [4]. Episodes of diarrhea can lead to subsequent ill health with the risk of complications being highly dependent on the infecting organism [5]. Alongside these potential health problems there are substantial social and economic costs [6], which intensify the need for improving surveillance and reducing the burden of intestinal infection. Knowing the location of incidents and identifying the causative organism facilitate better control and help target appropriate preventative measures.

Initial reporting by persons made ill, by owners/managers of a business involved, or by family doctors after seeing an ill patient is actively encouraged by staff from local authorities and Health Protection Units (HPUs), however, it is not mandatory and undoubtedly many outbreaks are missed. Some incidents may be recognized as clusters following an unexpected increase in laboratory isolates or after typing by a reference laboratory. In the Health Protection Agency North-East Region all outbreaks reported are recorded on a database held at both the Regional HPA Laboratory at Newcastle and the HPA commissioned laboratory at Middlesbrough. The present study seeks to examine data from outbreaks reported over a three-year period (2003 to 2005) to the laboratory at Middlesbrough. The aim is to study the outcomes and limitations of the present reporting system and to consider any options that might improve outbreak surveillance.

## 2. Experimental Section

### 2.1. The Database

The database is part of an intranet site operated by the HPA North-East region. Entry to the site is password protected and laboratory staff are responsible for entering the data onto the system. At present two laboratories are using the site with the system electronically generating the next available four digit number called the incident number (ILOG) when data has been entered and the screen prompt pressed. Details of an outbreak can be updated when more information becomes available by calling up the relevant ILOG number on the system. As well as the location of the incident, the name of the person notifying the outbreak, the local authority (unless within a hospital), the symptoms, and the date of onset are entered. The numbers of people thought to be exposed to the source (if known) together with those who were actually ill are also recorded. Whenever the source and/or the number of people exposed are unknown this is noted in a comments section on the ILOG form as a prompt for updating, if possible, at a later date. Each suspected outbreak is classified according to one of the following locations:- hospital, nursing or residential home, child care, adult day care, social occasion, school, retail premises, private residence, workplace and countryside. Space is also provided to record the causative organism. When the outbreak has stopped (either after closure by the outbreak team or after a period of one month without a specimen being submitted) the status is changed from ongoing to complete and the information is then filed. A search facility is built into the system and various entry points can be used to search the database.

Once an ILOG number has been set up the information is also entered into the microbiology laboratory computer system. This allows the ILOG number to be recorded at the time of specimen entry. The pathology computer system (APEX) has a search facility so that the results of all specimens logged to the outbreak can be viewed.

### 2.2. Reporting of Outbreaks

In this study an outbreak was defined as two or more cases of infectious intestinal disease linked by time and space. We excluded those outbreaks that were confined to persons living in the same household, unless the source was thought to be outside the home and other people might also have been exposed. Suspected outbreaks that took place in Tees Valley and County Durham (population approximately 1.1 million) or that occurred in patients in the James Cook University Hospital at Middlesbrough (acute hospital with approximately 1,000 beds) were included. Community outbreaks were reported to the laboratory by either CCDCs or nursing staff in a Health Protection Unit or by Environmental Health or Technical officers working for a local authority. In hospital, nurses in infection control or the Infection Control Doctor flagged up outbreaks. Early reporting was encouraged so that specimens are not lost to the system. When an outbreak was reported it provided an opportunity for the CCDC and microbiologist to discuss the likely pathogens and consider what pathogens should be looked for and the timing of results.

Reporting outbreaks to responsible authorities is voluntary. This means that investigation is patchy depending on both the action of those diagnosing or experiencing symptoms and on the number of persons ill and the severity of the symptoms. When alerted responsible authorities take action based on both the information received and on that gained by questioning those involved. The Consultant in Communicable Disease Control (CCDC) is responsible for setting up an Outbreak Control Team when more detailed investigation is needed either because of the severity or high number of cases or the need to establish control measures and prevent repeat episodes. The team includes as a minimum staff from the local authority, microbiology laboratory and the Health Protection Unit (HPU).

### 2.3. Microbiological Examination

For bacteriological tests the standard operating procedures described by the Heath Protection Agency were used. For faeces specimens the bacteria routinely looked for included *Salmonellas, Shigellas*, *Campylobacters*, and *Escherichia coli* O157, with other bacteria being added when foodborne or waterborne disease was suggested and the recorded symptoms and incubation period indicated that these might be important. As appropriate further identification and typing was performed at the HPA Centre for Infections in Colindale, London. Specimens were screened for crytosporidia by Auramine staining. Microscopic examination for other ova and cysts was performed on request. The enzyme immunoassay (EIA) kit from Premier was used to screen specimens for toxins A and B of *Clostridium difficile.* Testing for adenovirus (EIA Premier) astrovirus (EIA Dako), norovirus (EIA Dako), and rotavirus (EIA Dako) was carried out. Generally six specimens (when available) were tested for viruses, and whenever two or more patients were positive, no further specimens were screened. Faeces were referred to the Regional HPA Laboratory at Leeds for electron microscopy if no agent had been identified and an infectious cause was still suspected.

### 2.4. Routine Laboratory Surveillance

The results of positive cultures for gastrointestinal bacterial pathogens and positive tests for viruses obtained at Middlesbrough were routinely notified via the Co-Surv system to the Communicable Disease Surveillance Centre at Colindale, London. A search of this system identified how many of these organisms were reported by the laboratory during the three-year period. For noroviruses the time period was limited to April 2004 to December 2005 and this coincided with the introduction of the immunoassay system to detect this virus. Cross-reference to the pathology computer system (APEX) was used to determine whether or not follow up specimens had been sent for campylobacter and salmonella positives.

## 3. Results and Discussion

### 3.1. Case Reporting

Table 1 shows the organisms routinely reported by the Middlesbrough laboratory in the three-year study period. Of the 218 *Salmonella* isolates reported 61 were identified as being part of outbreaks. Although 813 *Campylobacters* were reported, none was part of an outbreak. Overall *C. difficile* toxins were the most common finding (1,092 reports), with most infections occurring in elderly care hospital wards or in nursing/residential homes. Laboratory investigation for viruses was limited to specific requests or suspected outbreaks.

### 3.2. Outbreaks Reported

At the time of this study outbreaks in the UK were not all routinely reported to national surveillance. Reporting was limited to laboratory-confirmed outbreaks (reported manually using a standard outbreak form) and to typing results, usually *Salmonellas* or *E. coli* O157, routinely sent from the reference laboratory to the national surveillance centre.

The highest monthly reports of enteric pathogens occurred during November and December 2004 and January 2005. The same seasonal affect was not as apparent in the two other years. The Winter peak was associated with a high incidence of norovirus. Although fewer in number, rotavirus infections were more likely to occur in the Spring, whereas in warmer months reports were more likely to be linked to foodborne transmission, notably *Salmonella*.

In 76 of the 279 suspected outbreaks (27.2%) no specimen was logged to the incident number (see Table 2). When specimens were received the median number per outbreak was 4, with a range of 1 to 121. In this study the detection of two or more patients in a suspected outbreak with an indistinguishable organism was considered sufficient to signify microbiological cause. Using this definition a microbial cause was found in 96 (34.4%) of the outbreaks. In over one-third of incidents (107/279; 38.4%) either none or only one positive specimen was found and no cause could be confirmed. Norovirus was the most common organism identified (53/279; 19.0%) with elderly patients in either hospital or nursing/residential homes being particularly at risk. Rotavirus was identified in 13 (4.7%) outbreaks with the elderly or very young being the most likely targets. *Salmonellas* were isolated in 15 outbreaks (5.4%), with 14 of these being linked to social events (wedding receptions or meals in hotels, restaurants or public houses). Three outbreaks were linked to *E. coli* O157 infection. More than one agent was found in six outbreaks (possibly due to the simultaneous detection of a past infection) and, of these, four involved *C. difficile* toxins and norovirus, one was *C. difficile* and rotavirus and the remaining one was norovirus and adenovirus.. Astrovirus was found in eight suspected outbreaks but in each case only one positive patient was identified and given the outbreak definition no outbreak linked to this virus was confirmed.

Almost half of the outbreaks identified (137/279; 49.1% see Table 2) occurred in residential/nursing homes. In 33 (24.1%) of these no specimens were received and in the others when specimens were submitted the median number per outbreak was 4, with a range of 1 to 17. With one exception (see 3.3 - outbreaks for which an outbreak control team was established) an outbreak control team was not set up for these incidents, however, a member of the HPU team did investigate, recommend control measures, as necessary, and produce a summary report. When a cause was established, the vast majority were due to norovirus, however, five (all in the Spring season) were associated with rotavirus. Fifty-one outbreaks were recorded in hospitals during the three year period. In 12 of these no specimens were submitted and in another 17 none or only one positive sample was identified. The median number of specimens received was 6 with a range from 2 to 121. Again norovirus was the most common cause (14 outbreaks), and *C. difficile* toxin (alone or in combination) was recorded in 9 outbreaks and rotavirus in three. Of 31 outbreaks linked to social occasions, salmonellas were identified as the most likely cause. One outbreak was due to *C. perfringen*s. The distribution of outbreaks for the other locations is given in Table 2. Of the 60 incidents recorded, in 25 no specimens were received and a cause was identified in 15 (25%) with *E. coli* O157 being found in three of them (see 3.3 - outbreaks for which an outbreak control team was established and Table 3).

Of the 279 outbreaks only 11 were identified as probably foodborne. In three more the evidence pointed towards contaminated food but no specific item could be identified. One outbreak was linked to untreated water and another two were associated with contaminated kitchen equipment.

### 3.3. Outbreaks in the Community for which an Outbreak Control Team was Established

During the study period outbreak control teams were established to investigate 21 outbreaks in the local community (Table 3). *Salmonella enteritidis* figured prominently and this species was responsible for 12 incidents. The largest was linked to a Chinese-style buffet restaurant (138 confirmed cases) and was the first phage type 56 outbreak to be reported in England. Two outbreaks, separated by a period of almost four months, and attributed to *S. enteritidis* phage type (PT) 14b was linked to the consumption of prawn toast from one supplier [7]. The product, supplied frozen and raw, contained imported egg and was positive for the same phage type. Eggs may have also been the source in a nursery outbreak of *S. enteritidis* PT4; however, person to person transmission was also evident among the children. Two outbreaks, separated by almost a year, occurred in one restaurant. Both were due to *S. enteritidis*, albeit of different phages types and both were thought to be linked to the preparation of tortillas using shell eggs. A recommendation to use pasteurized egg made after the first outbreak had not been implemented. Of the three *E. coli* O157 outbreaks, one was linked to a school visit to an open farm, one was associated with untreated water at a caravan site and the third was linked to the consumption of cooked meats from a butchers shop. In this case isolates from the raw meat scales and from the till keypad were indistinguishable from the human strains. The single outbreak due to *C. perfringens* was typical in that a large quantity of food, a chicken curry, was not properly reheated prior to serving. A strain, indistinguishable from the human cases, was isolated from a sample of the curry. An unusual outbreak linked to whipworm occurred in a small residential home for adults with severe learning difficulties. This may have been due to faecal contamination of the garden and subsequent eating of contaminated soil by some of the residents.

### 3.4. Discussion

This study used a database as a tool for the retrospective analysis of outbreaks. The database enabled all records to be held in one place with the facility that new information could be easily added. Linking the ILOG number to the laboratory computer system also enabled microbiology test results to be included. One limitation is that the system depends upon one or more individuals voluntarily notifying the authorities in order to begin the process and, given that most cases of gastroenteritis are self-limited this probably accounted for the low rate of ascertainment. The information is also not always timely thereby increasing the difficulty of obtaining relevant epidemiological information and collecting specimens from those that were ill. Wider distribution of the database beyond the small group of specialists currently involved may enhance the value of the system.

We found that very few specimens were submitted to the laboratory in relation to the number of people experiencing symptoms. In point-source outbreaks late reporting could account for fewer specimens being submitted, as many cases would have recovered prior to notification, however, person to person spread played a major part in many of the outbreaks described here and therefore timing was not the only reason for few specimens being received. Whereas sampling of all ill cases in large outbreaks might overwhelm limited laboratory resources, the evidence here suggests that, even in larger outbreaks, sampling rates remain low. We found that in more than a quarter of the outbreaks no sample was logged. In order to increase specimen submission, both persons ill and the general practitioners treating them should consider two questions. First, are there others with a similar illness occurring at the same time, and secondly, have foods been eaten outside the home in the previous seven days e.g. from a restaurant or take-away premises or after attending a social function where food was served. An affirmative answer to one of these questions should prompt collection of a specimen and the recording of this highly relevant information on the request form. In this study we did not have information on incidents that were confined to one household, however, we recognize that outbreaks in the domestic setting are undoubtedly common and contribute to the overall picture of outbreaks.

Initially we were surprised that so few foodborne outbreaks were identified. However, outbreaks in long-term care were the most common and the vast majority of these appear to be spread person to person rather than linked to food preparation or consumption. We cannot rule out, however, that food did not play some part in those outbreaks for which no foodborne link could be established. Stopping outbreaks in care facilities is particularly difficult and early recognition and prompt action is needed. Recent guidelines [8] emphasise key issues, notably a written policy (with access to advice at all times), isolation of infected residents, availability of additional care staff, good hand-washing practices, and early reporting to HPUs. It may well be that all these cannot be achieved within current resources and that, without substantial cost increases, these incidents remain very difficult to control effectively.

Using our outbreak definition of two or more laboratory-confirmed cases we identified a microbial cause in approximately one-third of the outbreaks reported. A recent one-year intensified study in The Netherlands [9] identified a pathogen in 54% of outbreaks. Their outbreak definition was the occurrence of diarrhoea and/or vomiting in at least five cases with some common factor. As here noroviruses were the most common cause. The higher rate of detection of noroviruses in the Dutch study (55.2% compared to 19% here) may be partly linked to a more sensitive detection method (PCR performed in the national reference institute compared to routine EIA in a clinical laboratory) and also to their special effort to examine stool specimens from at least five ill patients in each outbreak. The frequent association between noroviruses and outbreaks is linked to their high infectivity and the short-lived immunity to them [10]. Like others we found that *Campylobacters* were commonly identified pathogens but none was reported as part of an outbreak. The reasons for this are unclear as there is plenty of evidence showing that *Campylobacters* may be transmitted by a variety of foods, water and the environment, however, most infections, despite a relatively low infectious dose, remain sporadic or affect the members of one household only [11]. *C. difficile* was frequently isolated from cases of diarrhea, however, the high background levels, particularly in wards or homes for the elderly, can make it difficult to identify this organism as a cause of outbreaks [12]. It is clear, however, that *C. difficile* can cause serious outbreaks in debilitated patients [13]. In this study about one-third of routine salmonella notifications were proven to be part of outbreaks. Undoubtedly some outbreaks were missed e.g. a significant number of isolates were from people returning from holidays abroad, from whom no further details were available, and therefore the proportion of *Salmonella* infections matched to outbreaks is likely to be an underestimate.

This study supports the need for new surveillance systems to provide a more effective outbreak detection system [14]. Syndromic surveillance systems are being used to try and capture data from changes in behaviour that might indicate the early stages of an outbreak [15]. Unfortunately, because in most people, gastrointestinal infections are self-limiting and, symptoms disappear quickly, the majority do not change their behaviour in such a way that would trigger the surveillance system. However in some sections of the community, notably children, changes in practice are more likely to be seen.

Systems that utilize the speed and availability of the Internet are being introduced. An automated laboratory based system has been established in the Netherlands that is updated daily [16]. In the Program for Monitoring Emerging Diseases (ProMED-mail) [17], reports are screened, verified and then posted on the Internet. Rapid dissemination is also achieved via the Enter-net and Salm-gene databases [18]. All these systems are intended for international monitoring and depend on laboratory confirmation as the source of information. The Internet, however, has been used for rapid reporting of local outbreaks. The RUsick2 Foodborne Disease Forum is a web-based system that encourages people with illness to contact a website and record information about their illness. The site is always open, information can be updated at any time, and is claimed to be user friendly. Operators look for common links between patients and then notify appropriate local health professionals Measures are in place to control bogus reports that may be either deliberate or suggested by reading other entries. A pilot study, admittedly actively supported by a publicity campaign, showed more than a four-fold increase in reporting [19]. Recent government figures [20] indicate that nearly two-thirds of adults in the UK access the Internet. Those without web access could enter data via a receptionist at a doctor’s surgery, however, in practice this is less likely to happen. Perhaps one limiting factor is the newness of this approach with people being either unwilling to share information over the Internet or being concerned about the lack of human intervention. People may be embarrassed by a request for the collection of faeces for laboratory testing and, unless symptoms are particularly severe, would rather wait until better rather than involve the public health agencies or their local doctor.

## 4. Conclusions

This study has highlighted the limitations of the current identification system for general outbreaks of gastrointestinal disease in the Tees Valley and County Durham areas of England. In many incidents investigation was limited by the small number of specimens logged to an outbreak and in 76 of the 279 reported incidents no specimen was logged at all. In order to improve the recognition of outbreaks, both those that are ill and the general practitioners who are looking after them need to appreciate the importance of laboratory testing as part of outbreak investigation. Better control measures in care homes are needed. Inevitably the current systems fail to pick up some outbreaks and complementary approaches are needed to improve outbreak surveillance. The speed and availability of the Internet is suggested as one way of enhancing data collection.

## Figures and Tables

**Table 1.** Routine notifications of enteric pathogens by Middlesbrough Microbiology Laboratory, 2003–2005.

**Year**	**Organism**								

	**Campylobacter**	**Salmonella** (enteritidis)	**Shigella**	***C. difficile*** toxins	***E. coli*** O157	**Crypto** sporidium	**Astrovirus**	**Norovirus**	**Rotavirus**
**2003**	259	86 (57)	1	233	0	42	35	NA*	164
**2004**	258	56 (34)	2	327	1	19	34	53	120
**2005**	296	76 (48)	1	532	1	25	50	96	122
**Total**	813	218(139)	4	1092	2	86	119	149	406

* Not available in first year as only recorded after introduction of immunoassay method

**Table 2.** Etiological agents/organisms identified in reported outbreaks recorded in the ILOG system, Middlesbrough Microbiology Laboratory, 2003–2005.

**Location**	**Number**	**Specimens**	**Cause1**					
			Salmonella	*C. difficile*	Norovirus	Rotavirus	Other (state)	None found 2
**Hospital**	51	12	0	9	14	3	0	17
Residential Nursing	137	33	0	4	31	5	2 (*Trichuris*) (Adenovirus)	64
Child care	30	10	1	0	3	5	0	11
Adult day care	5	3	0	0		0	0	2
Social event	31	6	15	0	3	0	1	6
School	14	8	0	0	0	0	0	6
Retail premises	3	0	1	0	0	0	1 (*E. coli* O157)	1
Private Residence	2	0	0	0	2	0	0	0
Workplace	2	2	0	0	0	0	0	0
Other^3^	4	2	0	0	0	0	2 (both *E. coli* O157)	0
Total	279^4^	76	17	13	53	13	6	107

1 Agent/organism detected in two or more patients;

2 None found signifies that an agent was found in none or only one of the patients linked to the incident;

3 Location not matched to any of the other specified categories;

4 In 6 of the 279 outbreaks more than one agent was detected in two or more patients

**Table 3.** Community-based outbreaks reported to Middlesbrough Microbiology Laboratory for which an outbreak control team was established, 2003 to 2005.

**Location**	**Organism as(type)**	**Cases Confirmed**	**Cases At risk**	**Hypothesis Cause/contributory factor**
Restaurant	*S. typhimurium*	16	unknown	unknown
Buffet	*S.enteritidis* (56)	138	unknown	poor practices
Open farm	*E.coli* O157 (RDNC 1)	2	unknown	animal contact
Restaurant	*S.enteritidis* (4)	5	unknown	possible salad items
Tapas bar	*S.enteritidis* (1)	22	unknown	possible eggs
Restaurant	*S.enteritidis* (1)	10	unknown	unknown
Caravan park	*E.coli* O157 (21/28)	60	175	private water supply
Butchers	*E.coli* O157 (21/28)	11	unknown	cross contamination of cooked meats
Delicatessen	*S.typhimurium* (104)	107	unknown	possibly chicken
Tapas bar	*S.enteritidis* (4)	5	250	possible eggs
Adult care home	*Trichuris trichiura*	4	7	contaminated soil
Wedding reception	*S.enteritidis* (14b)	4	75	prawn toast
Concert	Norovirus	2	400	person to person
Buffet meal	*S.enteritidis* (14b)	6	93	prawn toast
Community	*Cryptosporidium parvum*	13	unknown	possible open farm visit
Buffet meal	*Clostridium perfringens*	4	120	chicken curry
Nursery	*S.enteritidis* (4)	13	120	possible egg and case to case
Take-away business	*S.enteritidis* (4)	4	unknown	eggs likely
Restaurant	*S.enteritidis* (4)	11	unknown	possible cross-contamination
Restaurant	*S.enteritidis (6)*	3	unknown	unknown
Take-away business	*S.enteritidis* (4)	6	unknown	eggs likely
